# Mimicking classical noise in ion channels by quantum decoherence

**DOI:** 10.1038/s41598-024-67106-6

**Published:** 2024-07-12

**Authors:** Mina Seifi, Ali Soltanmanesh, Afshin Shafiee

**Affiliations:** 1 Research Group on Foundations of Quantum Theory and Information, Department of Chemistry, Sharif University of Technology, P.O. Box 11365-9516, Tehran, Iran; 2 Philosophy of Science Group, Sharif University of Technology, Tehran, Iran

**Keywords:** Ion channels, Quantum decoherence, Classical noise, Spin–Boson model, Biophysical chemistry, Biological physics, Chemical physics, Quantum physics, Theoretical physics

## Abstract

The mechanism of selectivity in ion channels is still an open question in biology. Recent studies suggest that the selectivity filter may exhibit quantum coherence, which could help explain how ions are selected and conducted. However, environmental noise causes decoherence and loss of quantum effects. It is hoped that the effect of classical noise on ion channels can be modeled using the framework provided by quantum decoherence theory. In this paper, the behavior of the ion channel system was simulated using two models: the Spin–Boson model and the stochastic Hamiltonian model under classical noise. Additionally, using a different approach, the system’s evolution was modeled as a two-level Spin–Boson model with tunneling, interacting with a bath of harmonic oscillators, based on decoherence theory. We investigated under what conditions the decoherence model approaches and deviates from the noise model. Specifically, we examined Gaussian noise and Ornstein-Uhlenbeck noise in our model. Gaussian noise shows a very good agreement with the decoherence model. By examining the results, it was found that the Spin–Boson model at a high hopping rate of potassium ions can simulate the behavior of the system in the classical noise approach for Gaussian noise.

## Introduction

Ion channels are protein complexes that play an important role in the generation of electrical signals in many cellular processes of biological systems^1–4^. These channels regulate the flux of ions such as sodium and potassium with a highly selective conductance across the membrane^5^. Dysfunction of these channels is related to a wide range of diseases. Considering the wide role of ion channels in biological processes, the discovery of a mechanism that clearly describes the function of these channels is significant from a biological point of view. Selectivity is a fundamental feature of ion channels. In fact, each ion channel is responsible for the passage of a specific ion; for example, potassium channels allow only potassium ions to pass through while preventing the passage of other ions.

**Figure 1:**
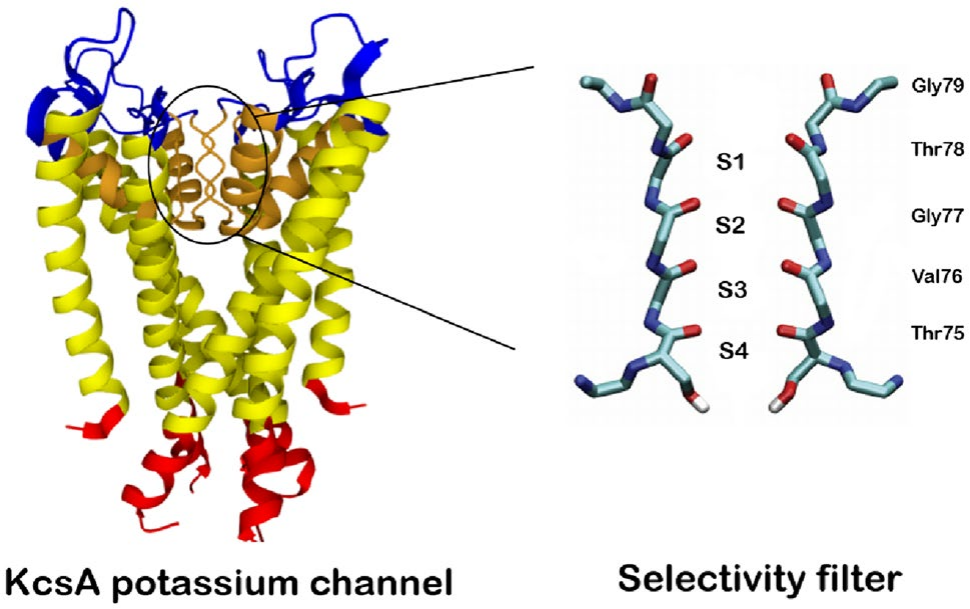
(Left) A KcsA potassium ion channel representation after PDB 1K4C. (Right) Two P-loop monomers in the selectivity filter, composed of the sequences of TVGYG amino acids [T (Threonine, Thr75), V (Valine, Val76), G (Glycine, Gly77), Y (Tyrosine, Tyr78), G (Glycine, Gly79)].

A selectivity filter (SF) is part of a protein that forms a narrow tunnel inside an ion channel that is responsible for selecting and rapidly directing specific ions across the cell membrane^6–8^. The KcsA channel is a well-known and widely studied ion channel. The structure of the selectivity filter in the KcsA potassium channel has been determined from the soil bacterium Streptomyces Lividans^9^. The length of the channel and its selectivity filter are 3.4 and 1.2 nm, respectively. The selectivity filter consists of four P-loops and has a structure similar to an alpha helix. Each P-loop consists of five amino acids linked by peptide units (H–N–C=O). Carbonyls are responsible for trapping and moving ions inside the filter. An overview of the KcsA channel structure and selectivity filter is shown in Fig. 1. Although the difference in the atomic radii of $\text {K}^{+}$ and $\text {Na}^{+}$ is only about 0.038 nm, the selectivity filter selects $\text {K}^{+}$ ions at a rate close to the diffusion limit ($10^{6}$–$10^{8}$ $\text {s}^{-1}$) with a ratio at least 10,000 times greater than that of sodium ions^10–12^. Understanding the precise physical mechanism responsible for this paradox of rapid conduction and high selectivity remains a significant challenge. Extensive research has been done to understand the selectivity mechanism of ion channels^13–21^. However, until now, most classical models have not been able to adequately explain ion selection at the nanoscale, and many problems remain unsolved. Given the inadequacy of classical mechanics in explaining ion selectivity, it seems that quantum mechanics should be used to address this issue.

To accurately describe quantum systems, it is necessary to study their interaction with their environment. The theoretical framework necessary to consider these interactions is provided by the theory of open quantum systems. Using this theory, a description of the quantum system coupled with the environment is provided, and then, within the framework of this mechanics, the evolution of the system is investigated by applying appropriate quantum operators. Many quantum systems can be studied using the theory of open quantum systems. Among others, we can mention the types of macroscopic quantum systems like biological systems, in some of which traces of quantum effects have been observed^22–31^.

Recently, it has been suggested that based on the time, size and energy scale of the transfer phenomena in ion channels, quantum coherence probably plays a role in the selection and transfer of ions^32–37^. However, due to the strong system-environment interaction, any coherence is likely short-lived. One can only speculate about the functional roles of coherence in ion channels, in which, there is still no precise experimental technique to provide information about the parameters used in effective models. The issue of the regime (quantum or classical) where these channels can be modeled best is a question that must be answered with the help of experience.

There are different theoretical frameworks for examining coherence in quantum systems^38^. A standard approach is to model the system and the environment as a large quantum system and track the effects of the environment (usually in the Born-Markov approximation) to obtain a single description of the system in question. An alternative approach is that instead of considering the system and its environment completely quantum, they consider the effect of the environment as a classical noise in the degrees of freedom of the system^39^. In this picture, quantum decoherence is simulated by averaging over a set of random but unitary quantum dynamics. This approach can often mimic standard open system dynamics and be used to simulate environmental effects.

It should be noted that there is a fundamental difference between decoherence and classical noise, and this difference is well-understood. In real decoherence, the unitary evolution of the system and the environment causes the non-unitary evolution of the reduced density matrix of the system. In the classic noise model, because the system is not coupled with any external environment, the dynamics of the system in each individual realization of the noise is unitary, so the loss of coherence is determined only in an ensemble. Despite the fundamental difference between classical noise and decoherence, the noise model can mimic the effects of decoherence well because both models reduce coherence.

The stochastic picture with classical noise has been widely used in physics and chemistry for various cases such as non-Markovian dynamics, many-body open quantum systems, and central spin problems^40–42^. Budini investigated the effect of classical random fields on quantum systems, specifically for studying the heating of trapped ions. He used a stochastic Hamiltonian for the system and by using averaged dynamics, he obtained a general description of the decoherence behavior of the system^43^. Chenu *et al.* presented a scheme for many-body decoherence simulation based on the unitary evolution of a stochastic Hamiltonian due to the addition of classical noise. This scheme can be readily implemented in various experimental platforms such as trapped ions and superconducting qubits^41^. Szańkowski and Cywiński formulated the necessary criteria for simulating the dynamics of open quantum systems with an external noise field that replaces the environmental degrees of freedom^44^. Crow and Joynt showed that the decoherence caused by the interaction of a qubit and a quantum bath can be classically simulated by the unitary evolution of a stochastic Hamiltonian^45^. Gu and Franco compared the quantum decoherence, which is caused by the entanglement of the system with its environment, with the apparent decoherence, which is obtained by averaging over an ensemble of unitary evolutions caused by a stochastic Hamiltonian, and presented the necessary conditions for quantitative modeling of decoherence by the classic noise picture^39^. Ma *et al.* used a Gaussian noise model for electron spin qubits in natural silicon and showed a good agreement between the results of the classical noise model with fully-quantum bath theory and experimental measurements to investigate the decoherence of this system^46^. The dynamics of quantum systems under quantum and classical noise were investigated by Saira *et al.* Quantum noise arises from the coupling of the microscopic system to its macroscopic environment, while classical noise is described by a random process that causes the time evolution of a closed quantum system. According to this analysis, fully quantum models can be depicted in the Born approximation with a quantum system under classical noise^47^. Schneider and Milburn considered white noise as random processes of the decoherence source for a trapped ion and presented a simple master equation of this source. The results of their investigation showed a good agreement with the recent experiments in terms of quality^48^.

In this work, the focus is on whether solving the ion channel problem considering the thermal bath is a correct assumption or not. The main question is, “When can classical noise be mimicked with quantum decoherence for the potassium ion channel?” This work has been done by examining the system in the presence of environmental noise and comparing the results with real decoherence. To investigate the quantum decoherence in this system, the Spin–Boson model has been used. Classical noise is modeled with a stochastic parameter in the Hamiltonian. In this work, we examine under what conditions the decoherence model approaches the noise model and moves away from the noise model. We will consider two commonly used noises, Gaussian noise and Ornstein-Uhlenbeck noise, in our model. Then, conditions are analyzed under which the quantum coherence dynamics of an ion channel can be simulated by a stochastic Hamiltonian operator, which is an alternative to environmental degrees of freedom.

The present paper is organized as follows. In “Classical noise formalism” section, we introduce the formalism of environmental noise and explain the resulting loss of coherence. In “Decoherence and Spin–Boson model” section, we review the decoherence theory and specifically the Spin–Boson model. In “Model and methods” section, we introduce the desired model for the ion channel and with the help of two Spin–Boson and classical noise models, we check the loss of coherence in our purpose system. Then, the results of the previous two sections are compared in detail in “Results and discussion” section. Finally, the paper in “Conclusion” section is concluded.

## Classical noise formalism

There are different strategies for modeling the dynamics of open quantum systems^49^. In this part, the classical noise approach is introduced for mimicking decoherence, whereby the Hamiltonian includes a dynamical stochastic variable. The effect of the environment in this approach is to introduce classical noise in the degrees of freedom of the system^39–41,50^. The fundamental difference between classical noise and decoherence is that in the decoherence mechanism, the system is entangled with the environment, and the phase relations are irreversibly lost from the system. By contrast, in classical noise representation, the entanglement of the environment system does not occur because the entanglement is a type of non-classical correlation^51^. In classical noise formalism, the decay of coherence can only be manifest in an ensemble. Because the system is not coupled with any environment, the dynamics of the system are completely unitary in each individual realization of the noise process. So the decay of coherence appears by averaging over an ensemble of stochastic but unitary dynamics. Since both noise and decoherence models effectively cause the loss of coherence, the decoherence model can mimic the classical noise. However, the fundamental conceptual differences between the two still remain. Classical noises are generally classified into Gaussian and non-Gaussian according to the form of their probability distribution. Gaussian noises are the most common and simplest types of noises. According to Marcinkiewicz, probability distributions with analytical generating functions are divided into two categories: Gaussian distributions and those with an infinite number of cumulants, with the Gaussian distribution being unique in having a finite number of cumulants^52^. This feature emphasizes the special nature of Gaussian distributions in statistical analysis and their ubiquitous presence in various physical systems. Unlike Gaussian distributions, which are fully described by only their first two cumulants (mean and variance), distributions with an infinite number of cumulants require an infinite number of cumulants to be fully specified. Cumulants are a set of statistical quantities used to describe the shape and structure of probability distributions. Distributions with an infinite number of cumulants often exhibit more complex behavior and are less tractable mathematically compared to Gaussian distributions.

Usually, any classical noise is specified by its autocorrelation or equivalent power distribution (noise spectrum)^42^. For Gaussian environments (bosonic and fermionic environments), the noise is completely determined by the autocorrelation function. Non-Gaussian noises are usually used for anharmonic environments (spin environments), and multi-time correlation functions are needed to characterize these noises. Recently, Kiely has modeled noise in quantum systems using a dynamic stochastic parameter in the Hamiltonian system^53^. He investigated the derivation of exact master equations for several different common noises. Using these exact master equations avoids the numerous iterations needed to obtain accurate mean dynamics. Specifically, the exact master equation for colored Gaussian noise is as follows^54^:

1 $$\begin{aligned} \frac{d}{dt}{\hat{\rho }}=-\dfrac{i}{\hbar }[{\hat{H}}_{0},{\hat{\rho }}] -\dfrac{1}{\hbar ^{2}}[{\hat{H}}_{1}(t),\int _0^t\text {d}s C(t-s)[{\hat{H}}_{1}(s),{\hat{\rho }}(s)]] \end{aligned}$$

where ${\hat{H}}_0$ and ${\hat{H}}_{1}$ are noise-free and “noisy” Hamiltonians, respectively and $C(t - s)$ is the correlation function. According to Wiener-Khinchin’s theory, spectral power density and correlation function are related and the spectral density is equal to the Fourier transform of the correlation function. Among the various types of noises, Gaussian white noise is the most common one. This noise is completely uncorrelated in time and has a flat power spectrum. Markovian dynamics processes can be examined using white noise fluctuations^41^. By considering the correlation function $C(t) =\alpha \delta (t)$ for this noise and the corresponding constant power spectrum $S(\omega ) =\dfrac{\alpha }{2\pi }$, master Eq. (1) simplifies to

2 $$\begin{aligned} \frac{d}{dt}{\hat{\rho }}=-\dfrac{i}{\hbar }[{\hat{H}}_{0},{\hat{\rho }}] -\dfrac{\alpha }{2\hbar ^{2}}[{\hat{H}}_{1}(t),[{\hat{H}}_{1}(t),{\hat{\rho }}(t)]] \end{aligned}$$

where $\alpha$ specifies the noise strength. This equation is obtained by averaging over stochastic classical noises. Using this exact master equation avoids many repetitions to obtain the average dynamics. In “4Model and methods” section, we will explain the details of using this master equation in our model.

Then to complete our discussion, we will use a validated approximate master equation for various classical noise sources and check our model using this master equation. To begin with, assume that the fluctuations in ${\hat{\rho }}_z$ are defined as $\Delta {\hat{\rho }}_z = {\hat{\rho }}_z - {\hat{\rho }}$. Using this definition and averaging the von Neumann equation $\frac{d}{dt}{\hat{\rho }}_z = -\frac{i}{\hbar } [{\hat{H}}, {\hat{\rho }}_z]$ the following exact dynamical equation for ${\hat{\rho }}$ is obtained:

3 $$\begin{aligned} \frac{d}{dt}{\hat{\rho }} = -\frac{i}{\hbar } [{\hat{H}}_0, {\hat{\rho }}] -\frac{i}{\hbar }\langle [z(t){\hat{H}}{_1(t)}, \Delta {\hat{\rho }}{_z(t)}]\rangle \end{aligned}$$

where *z*(*t*) is a real function for a given noise realization and for simplicity, $\langle \Delta {\hat{\rho }}{_z}\rangle = 0$ is used and it is assumed that $\langle z(t)\rangle = 0$. To understand the dynamics of fluctuations, from its definition, $\frac{\partial }{\partial t}\Delta {\hat{\rho }}{_z} = \dfrac{d}{dt}{\hat{\rho }}_z - \dfrac{d}{dt}{\hat{\rho }}$, when combined with von Neumann’s equation and Eq. (3), is used, which gives:

4 $$\begin{aligned} \begin{aligned} \frac{\partial }{\partial t}\Delta {\hat{\rho }}_z&+ \frac{i}{\hbar } [{\hat{H}},\Delta {\hat{\rho }}_z] \\&= -\frac{i}{\hbar } [z(t){\hat{H}}{_1(t)}, {\hat{\rho }}] + \frac{i}{\hbar }\langle [z(t){\hat{H}}{_1(t)},\Delta {\hat{\rho }}{_z(t)}]\rangle \end{aligned} \end{aligned}$$

The formal solution of this equation can be written as follows:

5 $$\begin{aligned} \Delta {\hat{\rho }}_{z}(t) = -\frac{i}{\hbar } \int _{0}^{t} ds\, U_{z}(t, s) \nonumber \\ \left\{ [z(s){\hat{H}}_{1}(s), {\hat{\rho }}(s)] - \left\langle [z(s){\hat{H}}_{1}(s),\Delta {\hat{\rho }}_{z}(s)]\right\rangle \right\} U_{z}^\dagger (t, s) \end{aligned}$$

where

6 $$\begin{aligned} U_{z}(t, s) = {\mathcal {T}} \exp \left[ -\frac{i}{\hbar } \int _s^t dt' \, H(t') \right] \end{aligned}$$

represents the time evolution operator and ${\mathcal {T}}$ denotes the Dyson time-ordering operator. At this point, Eq. (5) will be expanded to first order in ${\hat{H}}_1$ as:

7 $$\begin{aligned} \Delta {\hat{\rho }}{_z(t)} \approx -\frac{i}{{\bar{h}}} \int _{0}^{t} ds\, U_0(t, s)[z(s){\hat{H}}{_1(s)}, {\hat{\rho }}(s)]U_0^\dagger (t, s) \end{aligned}$$

**Figure 2:**
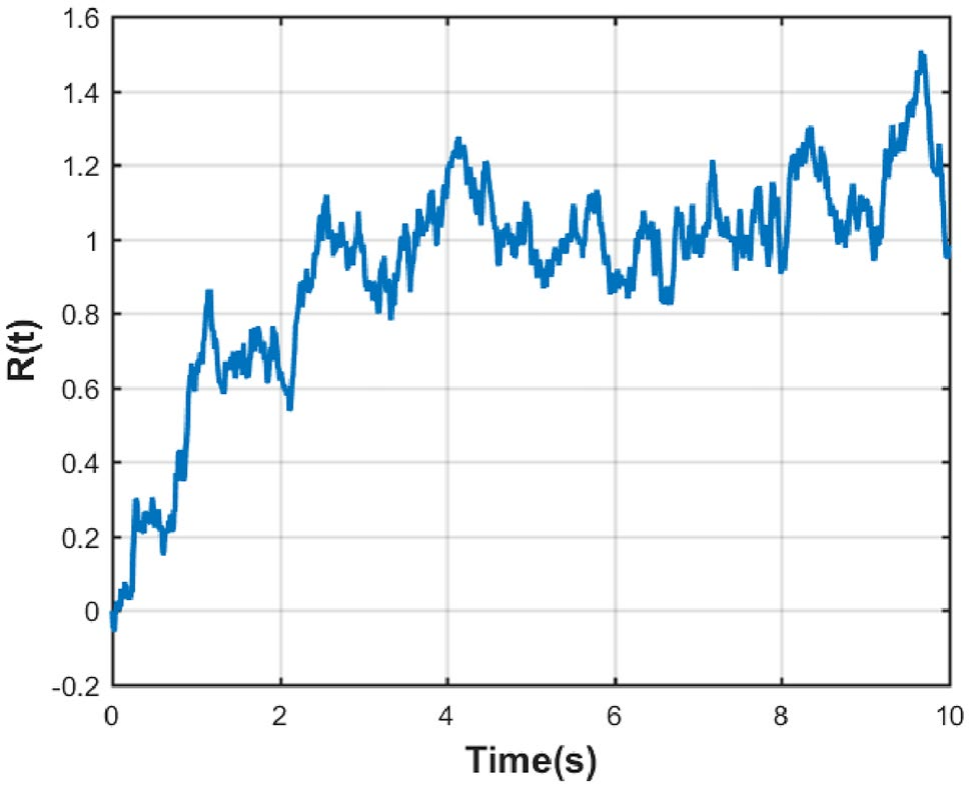
Ornstein-Uhlenbeck Process.

Placing this approximation into the precise master Eq. (3) results in an approximate master equation that is second-order in ${\hat{H}}_1$,

8 $$\begin{aligned} \begin{aligned} \frac{d}{dt}{\hat{\rho }}&= -\frac{i}{\hbar } [{\hat{H}}_0, {\hat{\rho }}] \\&\quad - \frac{1}{\hbar ^2}[ {\hat{H}}_1(t), \int _{0}^{t} ds\, C(t, s)U_0(t, s)[{\hat{H}}_1(s), {\hat{\rho }}(s)]U_0^\dagger (t, s)] \end{aligned} \end{aligned}$$

where $C(t, s) = \langle z(t)z(s) \rangle$. This master equation is applicable to any type of classical noise^53^, and we will specifically utilize it in our model in “4Model and methods” section, for Ornstein-Uhlenbeck noise, which is another common type of noise. Ornstein-Uhlenbeck noise, a stochastic process widely used in modeling systems with mean-reverting behavior, has unique features that make it well-suited for describing temporally correlated noise^55^. The Ornstein-Uhlenbeck process is stationary, Markovian, and has finite variance. It is often used to model systems with a tendency to revert to a mean value over time. Its mathematical model typically involves a differential equation with both deterministic and stochastic components. The deterministic term drives the process toward the mean, while the stochastic term introduces random variations. Ornstein-Uhlenbeck noise extends beyond Gaussian noise by incorporating temporal correlation into its framework. This noise plays an important role in simulating and understanding complex systems in modeling real and temporally correlated fluctuations. Due to these properties, it enjoys broad applicability across various domains such as physics, finance, neuroscience, and biology, serving as a valuable tool for capturing realistic noise dynamics. In Fig. 2, you can see the sample path of the Ornstein-Uhlenbeck process.

## Decoherence and Spin–Boson model

Every realistic system inevitably interacts with its environment^56–59^. For nanoscale quantum systems and quantum biological systems, this interaction is not insignificant; consequently, they should be considered to be open systems^60,61^. So, for the dynamic modeling of quantum biological systems, one should go to the theory of open quantum systems. By using decoherence theory and open quantum systems, a realistic picture of a distinct model can be obtained. Due to the manifold interactions between ions and multiple degrees of freedom of the channel, the dynamics of ion transport in the ion channel are very complex. In ion channels, rapid decoherence is apparently inevitable due to the strong coupling of the quantum state of the passing ion with the molecular vibrational modes of the environment of the protein. However, the time of the passage of the ion through the membrane is also very short (about 10–20 ns)^62^. The ratio of the time scale of the decoherence and the traversal time plays the main role in the understanding of maintaining coherence^63^. To maintain the quantum superposition of the transiting ion state, the decoherence time must be longer than this transit time. Based on this, it is suggested that the ion selectivity filter probably has quantum coherence. Note that for more rigorous conclusions, one should wait for more detailed simulations and experimental research. In the interaction between the system and its environment, since the degrees of freedom of the environment are infinite, the system cannot be characterized by specific states or a wave function. Therefore, a density matrix should be attributed to the system and its evolution should be checked over time. Each element of the main diagonal of the density matrix gives the probability of finding the system at a certain energy level^60^. Due to the dissipative terms of the master equation, the time evolution of open systems is non-unitary in contrast to closed systems. The Spin–Boson model has been extensively used in the literature to study dissipation and quantum decoherence. Recently, this model has been used in biological systems to investigate quantum coherence^64–68^. In this model, a single two-level system, e.g., a spin 1/2 particle, is coupled with a boson environment of harmonic oscillators. In the Spin–Boson model with tunneling, the general form of the Hamiltonian is defined as follows:

9 $$\begin{aligned} {\hat{H}}=\frac{1}{2}\omega _{0}{\hat{\sigma }}_{z}-\frac{1}{2}\Delta _{0} {\hat{\sigma }}_{x}+\sum _i(\frac{1}{2m_{i}}{\hat{p}}^{2}_{i} +\frac{1}{2}m_{i}\omega ^{2}_{i}{\hat{q}}^{2}_i)+{\hat{\sigma }}_{z}\otimes \sum _i c_{i}{\hat{q}}_{i} \end{aligned}$$

where $\Delta _0$ is the tunneling matrix element and represents the tunneling between two quantum states and $c_i$ is the coupling strength of the system-environment. An appropriate approach to formulating open quantum systems dynamics is to use quantum-dynamical equations, commonly known as quantum master equations (QME). The general expression of the Born-Markov master equation for the Spin–Boson model is obtained as^60^:

10 $$\begin{aligned} \frac{d}{dt}{\hat{\rho }}_s(t)=&-\dfrac{i}{\hbar }[{\hat{H}}^{'}_{s},{\hat{\rho }}_{s}(t)] -D[{\hat{\sigma }}_{z},[{\hat{\sigma }}_{z},{\hat{\rho }}_{s}(t)]] \nonumber \\&+\zeta {\hat{\sigma }}_{z}{\hat{\rho }}_{s}(t){\hat{\sigma }}_{y} +\zeta ^{*}{\hat{\sigma }}_{y}{\hat{\rho }}_{s}(t){\hat{\sigma }}_{z}, \end{aligned}$$

where

11 $$\begin{aligned} {\hat{H}}^{'}_{s}=[-\frac{1}{2}\Delta _{0}-\zeta ^{*}]{\hat{\sigma }}_{x} \end{aligned}$$

is the renormalized (“Lamb-shifted”) Hamiltonian of the system. The coefficient $\zeta$ is provided by

12 $$\begin{aligned} \zeta =\int _0^{\infty }\text {d}\tau [\nu (\tau )-i\eta (\tau )]\sin (\Delta _{0}\tau )\equiv f-\text {i}\gamma \end{aligned}$$

The coefficients D, f and $\gamma$ in the above equations are determined as follows:

13 $$\begin{aligned} D=\int _0^{\infty }\text {d}\tau \nu (\tau )\cos (\Delta _{0}\tau ), \end{aligned}$$

14 $$\begin{aligned} \textit{f}=\int _0^{\infty }\text {d}\tau \nu (\tau )\sin (\Delta _{0}\tau ), \end{aligned}$$

15 $$\begin{aligned} \gamma =\int _0^{\infty }\text {d}\tau \eta (\tau )\sin (\Delta _{0}\tau ), \end{aligned}$$

where

16 $$\begin{aligned} \nu (\tau )=\int _0^{\infty }\text {d}\omega J(\omega )\coth (\dfrac{\omega }{2k_{B}T})\cos (\omega \tau ) \end{aligned}$$

and

17 $$\begin{aligned} \eta (\tau )=\int _0^{\infty }\text {d}\omega J(\omega )\sin (\omega \tau ) \end{aligned}$$

are the noise and dissipation kernels, respectively^69^. Here $J(\omega )$ is the spectral density of the environment and the coupling between the system and the environment is described by it. In this work, an ohmic environment with a Lorentz-Drude cutoff frequency which has high applications, is used. For such environments, the spectral density is written as^70–72^:

18 $$\begin{aligned} J(\omega )=\dfrac{2M\gamma _{0}}{\pi }\omega \dfrac{\omega _{c}^{2}}{\omega _{c}^{2}+\omega ^{2}} \end{aligned}$$

where $\omega _{c}$ is the cutoff frequency and $\gamma _{0}$ constant specifies the effective coupling strength between the system and its environment. The first term to the right of Eq. (10) shows the unitary evolution of the density matrix. The second term, which is related to the interaction of the system with the environment, shows decoherence effects and the third and fourth terms describe the decay of the two-level system. In this work, the evolution of the reduced density matrix of the system has been investigated using the master Eq. (10), which is described in detail in the next section.

## Model and methods

**Figure 3:**
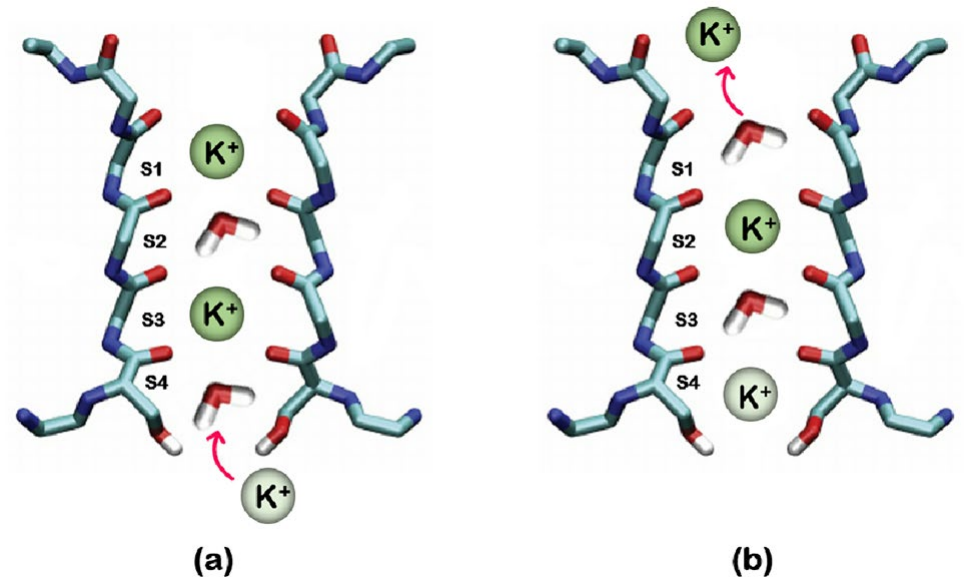
Schematic illustration of how a single file is transported through the KcsA ion channel selectivity filter. The two ions move in a concerted manner between two states, (**a**) 1,3 state and (**b**) 2,4 state, until a third ion comes in, shifting the ion to the other side of the queue.

Since the width of the selectivity filter is only about 0.3 nm, potassium ions must leave their hydrated shell before entering the selectivity filter and pass-through this filter in a single file^73–75^. The crystal structure of the KcsA potassium channel showed that the selectivity filter has four binding sites for $\text {K}^{+}$ (labeled with S1 to S4, from extracellular to intracellular) Fig. 1. In order to study the ion permeation mechanism via the selectivity filter, different experimental techniques like radiotracer flux essays and X-ray crystallography, and various computational methods such as molecular dynamics (MD) simulations have been used. The experiments conducted provide support for two mechanisms: “hard-knock” in which water molecules are ignored, and “knock-on” in which water molecules can be either present or absent. To determine the permeation model that is more consistent with the experiment, 2D IR spectra were calculated for all ion configurations using MD simulations. It was found that the knock-on model (with water molecules) is in good agreement with the experiment^76^. The knock-on model was first proposed in 1955 by Hodgkin and Keynes^77^. The ion transfer in this process is such that the selectivity filter is occupied by two potassium ions within a single file simultaneously with intervening water molecules that oscillate harmoniously between configurations 1 and 3 or 2 and 4 Fig. 3. Then the third $\text {K}^{+}$ ion enters from one side and the sequence of ion-water shifts, and finally, the potassium ion is displaced from the opposite side^78^. Notably, a more complex model that incorporates several states, including those with K atoms and vacancies (absence of $\text {H}_2\text {O}$), could be considered. However, such an expansion would increase the dimensions of the problem and potentially compromise the solution’s accuracy. Nonetheless, we anticipate the system’s overall dynamics to remain unchanged. Additionally, the stability of configurations 1,3 and 2,4 is confirmed. Hence, we concentrate on these two states to validate our model^9^. Furthermore, the Coulomb interaction proves highly effective on a prolonged time scale; therefore, we analyze this model within a relatively shorter time frame. Given the significant fluctuations of K+ ions within the ion channel, strong Coulombic repulsion probably does not manifest on this scale. So our model is based on the two described configurations. Therefore, we investigate an ion channel with four sites as shown in Fig. 3. In this model, we define a two-state system: (1) $\text {K}^{+}$ ions on the 1 and 3 sites and water molecules on the 2 and 4 sites, and (2) $\text {K}^{+}$ ions in the 2 and 4 states and water molecules in the 1 and 3 sites. Jumping from one site to another is associated with an energy barrier. This system can be shown in a two-dimensional Hilbert space with two states of $\vert 0\rangle$ and $\vert 1\rangle$ similar to a spin-1/2 system. In the following stages, we used two Spin–Boson and classical noise models to investigate the decoherence rate in the ion channel. First, using the Spin–Boson model and master Eq. (10), the evolution of the density matrix was obtained. $\hslash$ is supposed to be 1 in the rest of the paper.

In this model, the total Hamiltonian is defined as:

19 $$\begin{aligned} {\hat{H}}={\hat{H}}_s+{{\hat{H}}_\varepsilon }+{\hat{H}}_{int} \end{aligned}$$

Here

20 $$\begin{aligned} {\hat{H}}_s=\dfrac{1}{2}\omega _{0}\hat{\sigma _{z}}-\dfrac{1}{2}\Delta _{0}\hat{\sigma _{x}} \end{aligned}$$

is the self-Hamiltonian of the system. The asymmetry energy $\omega _0$ represents the energy difference between the basis states of the system( the eigenstates of z).

${\hat{H}}_\varepsilon$ represents the self-Hamiltonian of the environment which is composed of harmonic oscillators with frequency $\omega$. ${\hat{H}}_{int}$ describes the linear coupling between the z-coordinate of the system and the position of the coordinates of each environmental harmonic oscillator. According to the above discussion and considering the following equation for

21 $$\begin{aligned} {\hat{H}}^{'}_{s}=\dfrac{1}{2}\omega _{0}\hat{\sigma _{z}}-\dfrac{1}{2} \Delta _{0}\hat{\sigma _{x}}-\zeta ^{*}\hat{\sigma _{x}}\end{aligned}$$

Equation (10) can be rewritten in the basis of the eigenstates of the $\hat{\sigma _{z}}$ operator as:

22 $$\begin{aligned} \dfrac{d}{dt}{\hat{\rho }}_{00}=&\dfrac{i}{2}\Delta _0({\hat{\rho }}_{10}-{\hat{\rho }}_{01})+2\gamma {\hat{\rho }}_{01} \nonumber \\ \dfrac{d}{dt}{\hat{\rho }}_{01}=&\dfrac{i}{2}\Delta _0({\hat{\rho }}_{11}-{\hat{\rho }}_{00})+2i\zeta ^{*}{\hat{\rho }}_{11}-2if{\hat{\rho }}_{00}-(i\omega _{0}-4D){\hat{\rho }}_{01} \nonumber \\ \dfrac{d}{dt}{\hat{\rho }}_{10}=&\dfrac{i}{2}\Delta _0({\hat{\rho }}_{00}-{\hat{\rho }}_{11})+2i\zeta ^{*}{\hat{\rho }}_{00}-2if{\hat{\rho }}_{11}+(i\omega _{0}-4D){\hat{\rho }}_{10} \nonumber \\ \dfrac{d}{dt}{\hat{\rho }}_{11}=&\dfrac{i}{2}\Delta _0({\hat{\rho }}_{01}-{\hat{\rho }}_{10})+2\gamma {\hat{\rho }}_{10} \end{aligned}$$

With the condition that $J(\omega )$ is odd, $\gamma$ and *D* coefficients are simplified to get

23 $$\begin{aligned} \gamma =\dfrac{\pi }{2}J(\Delta _{0}) \end{aligned}$$

and

24 $$\begin{aligned} D=\dfrac{\pi }{2}J(\Delta _{0})coth(\dfrac{\Delta _{0}}{2k_{B}T}) \end{aligned}$$

In the high-temperature limit $\beta \omega _{c}\ll 1$( where $\beta =\dfrac{1}{k_{B}T})$, $coth(\dfrac{\beta \omega }{2})\thickapprox 2(\beta \omega )^{-1}$ and

25 $$\begin{aligned} f=2M\gamma _{0}k_{B}T\dfrac{\omega _{c}\Delta _{0}}{\Delta _{0}^{2}+\omega _{c}^{2}} \end{aligned}$$

Now, we employ the classical noise formalism to investigate our model using master Eqs. (2) and (8). To begin, consider the following total Hamiltonian

26 $$\begin{aligned}{} & {} {\hat{H}}(t)={\hat{H}}_0+z(t){\hat{H}}_{1} \end{aligned}$$

27 $$\begin{aligned}{} & {} {\hat{H}}_{0}=\dfrac{1}{2}\omega _{0}\hat{\sigma _{z}} -\dfrac{1}{2}\Delta _{0}\hat{\sigma _{x}} \end{aligned}$$

28 $$\begin{aligned}{} & {} {\hat{H}}_{1}=\hat{\sigma _{z}} \end{aligned}$$

where ${\hat{H}}_0$ is the noise-free Hamiltonian, ${\hat{H}}_{1}$ describes the “noisy” Hamiltonian, as previously introduced, *z*(*t*) is a real function for a given noise realization and it has replaced the interaction of the system-environment. Therefore, the master Eq. (2) for Gaussian white noise can be rewritten as follows:

29 $$\begin{aligned} \dfrac{d}{dt}{\hat{\rho }}_{00}=&\dfrac{i}{2}\Delta _0({\hat{\rho }}_{10}-{\hat{\rho }}_{01}) \nonumber \\ \dfrac{d}{dt}{\hat{\rho }}_{01}=&\dfrac{i}{2}\Delta _0({\hat{\rho }}_{11}-{\hat{\rho }}_{00}) -i\omega _{0}{\hat{\rho }}_{01}-2\gamma {\hat{\rho }}_{01} \nonumber \\ \dfrac{d}{dt}{\hat{\rho }}_{10}=&\dfrac{i}{2}\Delta _0({\hat{\rho }}_{00}-{\hat{\rho }}_{11}) +i\omega _{0}{\hat{\rho }}_{10}-2\gamma {\hat{\rho }}_{10} \nonumber \\ \dfrac{d}{dt}{\hat{\rho }}_{11}=&\dfrac{i}{2}\Delta _0({\hat{\rho }}_{01}-{\hat{\rho }}_{10}) \end{aligned}$$

Finally, we investigated master Eq. (8) for Ornstein-Uhlenbeck noise in our model. The correlation function for this noise is $C(t) = \dfrac{\alpha '}{2 \tau } e^{-\frac{|t|}{\tau }}$, where $\alpha '$ represents the strength of the noise and $\tau$ denotes the correlation time. So, considering Eqs. (27) and (28), the master Eq. (8) for Ornstein-Uhlenbeck noise can be rewritten as follows:

30 $$\begin{aligned} \begin{aligned} \frac{d}{dt}{\hat{\rho }}_{00} =&-\frac{i\Delta _0 {\hat{\rho }}_{01} }{2} + \frac{i\Delta _0 {\hat{\rho }}_{10} }{2} \\&\quad + \frac{4\alpha ' {\hat{\rho }}_{01} \exp \left( -\frac{it\Delta _0}{2} \right) \exp \left( -\frac{it\omega _{0} }{2} \right) \exp \left( -\frac{t}{\tau } \right) }{i\Delta _0 \tau + 2 + i\tau \omega _{0} } \\&\quad + \frac{4\alpha ' {\hat{\rho }}_{10} \exp \left( \frac{it\Delta _0 }{2} \right) \exp \left( \frac{it\omega _{0} }{2} \right) \exp \left( -\frac{t}{\tau } \right) }{i\Delta _0 \tau - 2 + i\tau \omega _{0} } \\ \frac{d}{dt}{\hat{\rho }}_{01} =&-i\omega _{0}{\hat{\rho }}_{01} - \frac{i\Delta _0 {\hat{\rho }}_{00} }{2} + \frac{i\Delta _0 {\hat{\rho }}_{11} }{2} \\ \frac{d}{dt}{\hat{\rho }}_{10} =&i\omega _{0}{\hat{\rho }}_{10} + \frac{i\Delta _0 {\hat{\rho }}_{00} }{2} - \frac{i\Delta _0 {\hat{\rho }}_{11} }{2} \\ \frac{d}{dt}{\hat{\rho }}_{11} =&2 \int \left( \frac{\alpha ' {\hat{\rho }}_{01} \exp \left( \frac{it\Delta _0 }{2} \right) \exp \left( -\frac{it\omega _{0} }{2} \right) \exp \left( -\frac{t}{\tau } \right) }{\tau } \right. \\&\quad \left. - \frac{\alpha ' {\hat{\rho }}_{10} \exp \left( -\frac{it\Delta _0 }{2} \right) \exp \left( \frac{it\omega _{0} }{2} \right) \exp \left( -\frac{t}{\tau } \right) }{\tau } \right) dt \\&\quad + \frac{i\Delta _0 {\hat{\rho }}_{01} }{2}- \frac{i\Delta _0 {\hat{\rho }}_{10} }{2} \end{aligned} \end{aligned}$$

**Figure 4:**
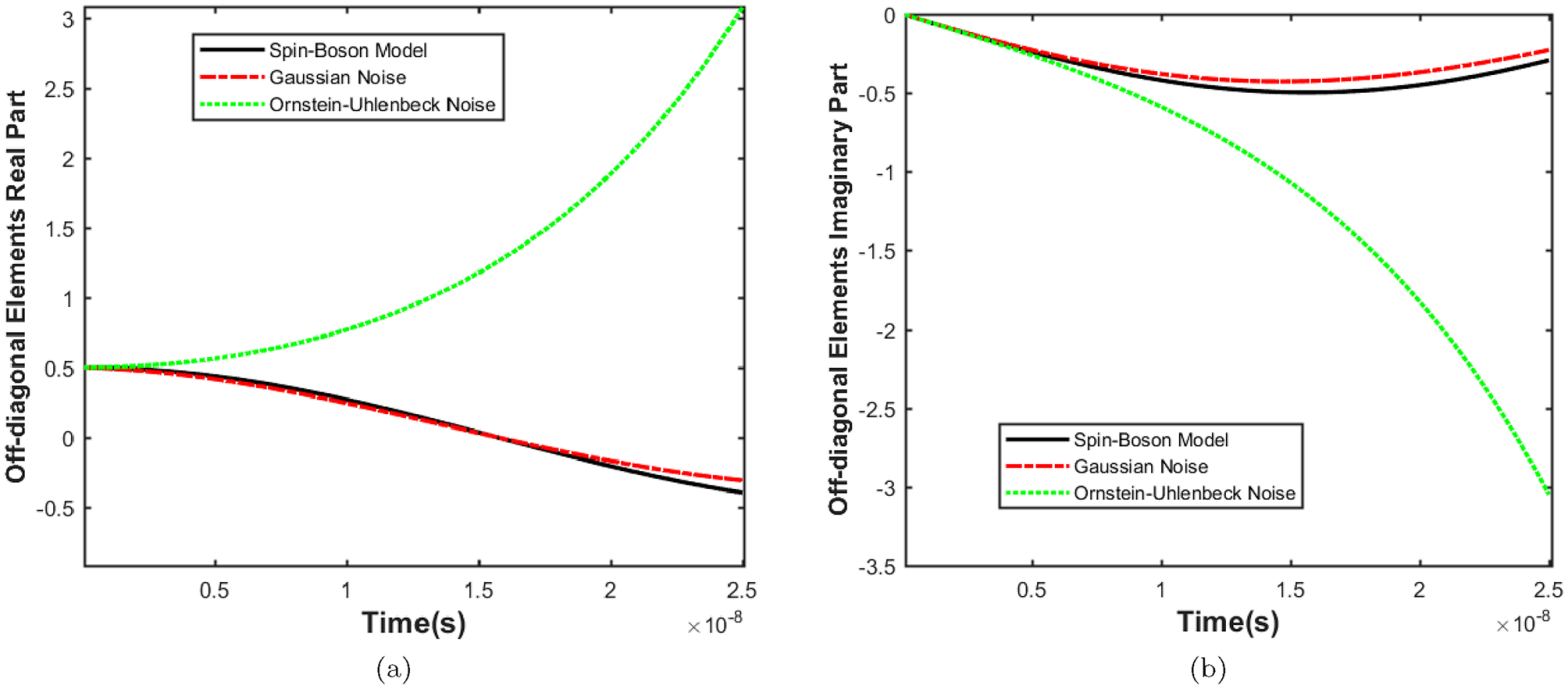
The evolution of off-diagonal elements of density matrix versus time with $\gamma =0.5\times 10^{7}s^{-1}$ and $\Delta _0=1\times 10^{7}s^{-1}$, (**a**) real parts and (**b**) imaginary parts.

**Figure 5:**
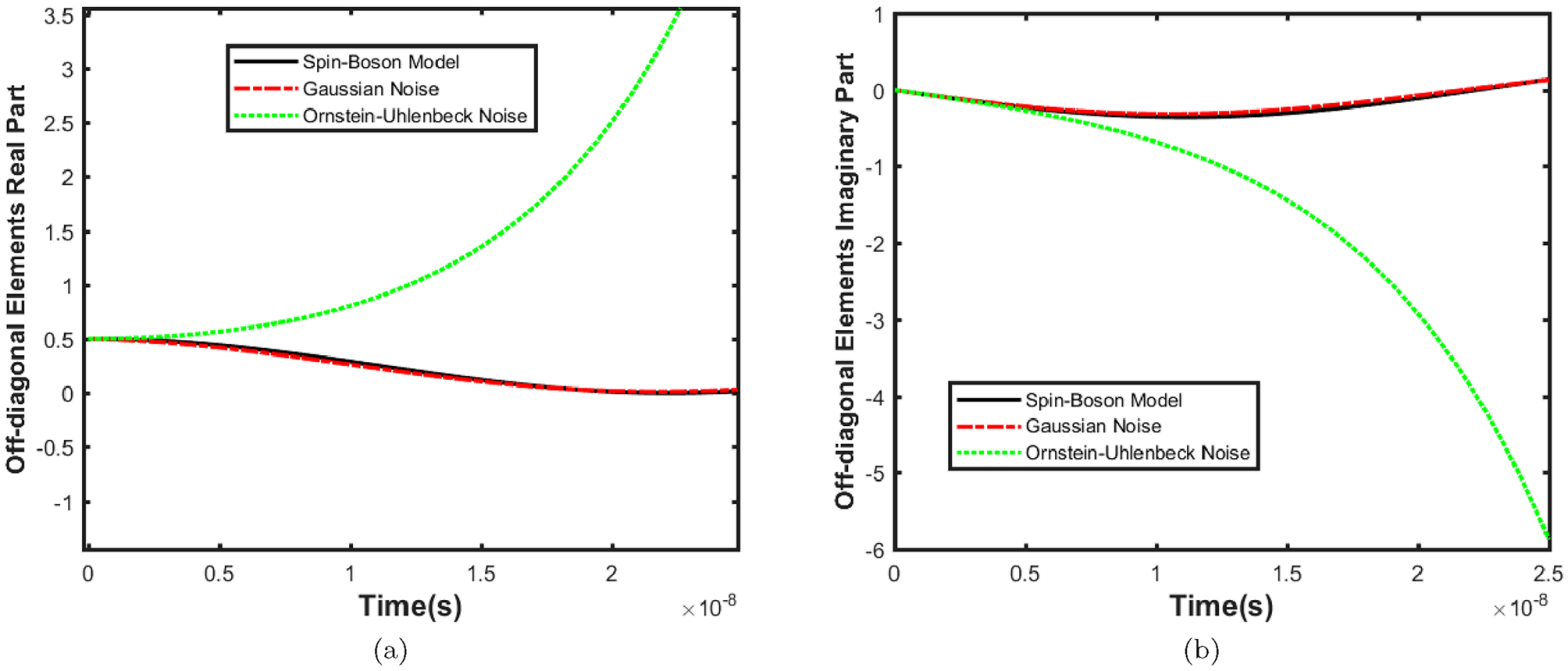
The evolution of off-diagonal elements of density matrix versus time with $\gamma =0.5\times 10^{7}s^{-1}$ and $\Delta _0=1\times 10^{8}s^{-1}$, (**a**) real parts and (**b**) imaginary parts.

**Figure 6:**
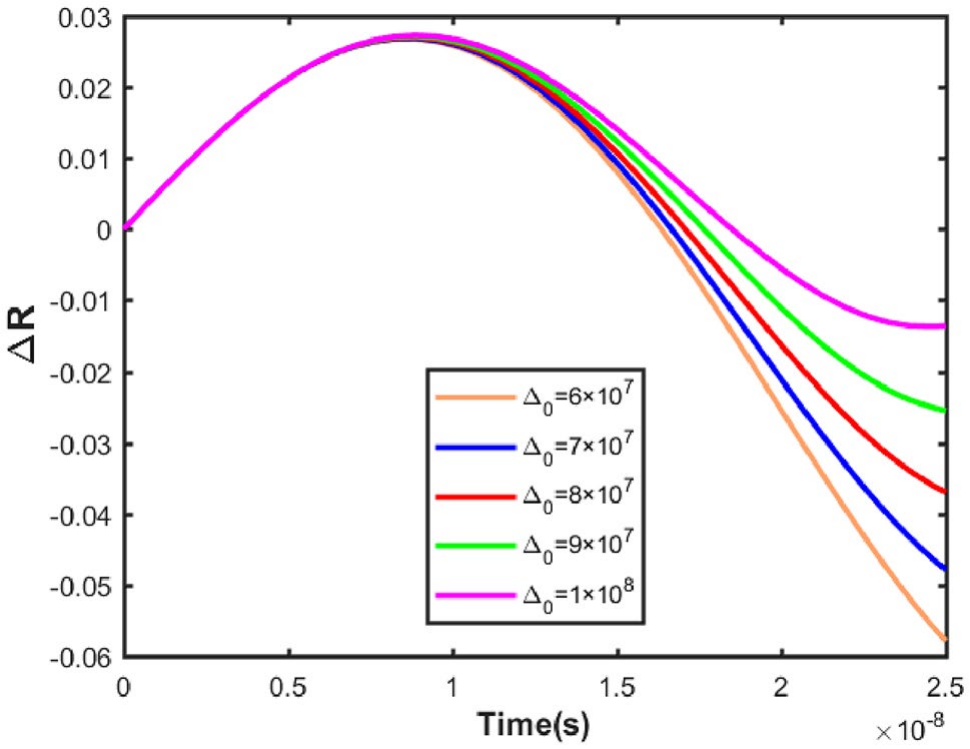
The changes of the $\Delta R$ parameter versus time with $\gamma =0.5\times 10^{7}s^{-1}$ in a variety of hopping rates $\Delta _0$ for Gaussian noise.

## Results and discussion

Recently, it has been suggested that hopping in ion channels may remain coherent during the process^33–36^. This coherence can play an important role in explaining ion channels’ selectivity and ion conduction mechanism. However, based on the quantum mechanical model, this system is expected to rapidly couple with an environment and pass through a decoherence process. Despite the existence of interaction between the channel and the protein environment, quantum coherence is expected to be maintained for shorter time scales than decoherence and play a vital role in the dynamics. Therefore, in order for the ions to maintain their quantum states during the mechanism of passing through these channels, the decoherence time must be longer than the time required for each ion to pass through the channel. To investigate the problem, first, the system of Eq. (10) was solved by considering the initial state of the system as the following superposition:

31 $$\begin{aligned} \vert \psi _i\rangle =\frac{1}{\sqrt{2}}(|0\rangle +|1\rangle ) \end{aligned}$$

Using thermal Broglie wavelength $\lambda _{dB}=1/\sqrt{2mk_BT}$, the order of decoherence in this problem can be obtained at a glance. For the decoherence time one gets

32 $$\begin{aligned} \tau _D=\frac{\Delta X^2}{\gamma \lambda _{dB}^2} \end{aligned}$$

where $\Delta X$ represents the dispersion in position space and

33 $$\begin{aligned} \gamma =\gamma _0\omega {\bar{n}}\frac{r^2}{1+r^2} \end{aligned}$$

with $r=\Lambda /\omega$ and ${\bar{n}}=(e^{\omega /k_BT}-1)^{-1}$. $\Lambda$ is the cut-off frequency and ${\bar{n}}$ demonstrates the mean environmental population based on Temperature (*T*).The value of the diffusing rate strongly depends on the frequency of the particles. The decoherence time for this ion channel based on the selection of the appropriate diffusion rate ($1\times 10^{6}s^{-1}$ to $1\times 10^{8}s^{-1}$) and its appropriate frequency ($1\times 10^{8}s^{-1}$ to $1\times 10^{12}s^{-1}$) varies between $1\times 10^{-10}s$ to $1\times 10^{-7}s$^33,34^. Considering the selectivity filter as a two-level system and using the Spin–Boson model, the behavior of the system in the interaction with the protein environment was investigated. The coupling between the system and the environment was characterized by the spectral density $J(\omega )$. To check the results of the two models, Eqs. (22), (29), and (30) are solved numerically. As mentioned, the hopping rate in the potassium ion channel is high and is of $10^{6}$-$10^{8}$ $\text {s}^{-1}$ order. Therefore, the compatibility of these two models is checked at different speeds. Off-diagonal elements show similar behavior, and the probabilities of these elements can be used to check the behavior of the system’s coherence. Here the evolution of the second element is presented as an example. First, these two models were considered at lower speeds, and the results are shown in Fig. 4 for illustration. This figure demonstrates that quantum decoherence results diverge from classical noise model results at longer times for both Gaussian and Ornstein-Uhlenbeck noises. The investigation of the Spin–Boson and classical noise models at higher speeds is presented in Fig. 5. Figure 5 illustrates that as the ion channel hopping rate increases, there is a strong agreement between the Spin–Boson and classical noise models for Gaussian noise. By comparing Figs. 4 and 5, it is evident that as the ion’s transit speed through the channel increases, the results of the Spin–Boson model come closer to the classical noise formalism for Gaussian noise, showing good agreement. In contrast, for Ornstein-Uhlenbeck noise, changes in the hopping rate have an insignificant impact on the results. Figures 5 and 5 illustrate that the Ornstein-Uhlenbeck noise does not match the Spin–Boson model, regardless of the hopping rate. This indicates that the quantum decoherence model in the ion channel cannot accurately mimic the Ornstein-Uhlenbeck noise model. This discrepancy is understandable because the speed of ions passing through the channel is high. Consequently, due to this high speed, noise changes do not occur on a timescale where the Markovian assumption is crucial. In this context, the noise can be considered almost instantaneous, making the Markovian nature of the noise less relevant. As a result, Ornstein-Uhlenbeck noise is not an appropriate model for comparison with the Spin–Boson model. Given the high hopping rate in the ion channel and based on Figs. 4 and 5, Gaussian noise appears to be suitable for our system. On the other hand, ion channel interactions are sharp interactions, so we expect that the appropriate distribution for our investigation is a sharp distribution. The Ornstein-Uhlenbeck distribution, being more diffuse than the Gaussian distribution, is not suitable for our model. Additionally, the Ornstein-Uhlenbeck distribution changes over time. Given the high speed of ion channel interactions, we do not expect the environment to change due to the interaction, making the Markov assumption less applicable in this context. Therefore, since the potassium ion channel must be investigated at high speeds, Gaussian noise is appropriate. In the following, for a more detailed analysis of the results, we defined the $\Delta R$ parameter as follows

34 $$\begin{aligned} \Delta R=R_{SB}-R_{N} \end{aligned}$$

where $R_{SB}$ and $R_{N}$, respectively, show the real part of the evolution of the elements off-diagonal of the density matrix for the Spin–Boson model and the noise formalism. $\Delta R$ parameter shows the difference between the results of two Spin–Boson and classical noise models. Therefore, in the following, we use this parameter to compare the results of these models. The results are checked in the range of the speed of the ions passing through the channel. The difference between the results of the two models at different speeds is investigated using Eq. (34). The results of this equation comparing the Gaussian noise model with the Spin–Boson model are shown in Fig. 6. Figure 6 shows that the difference between the Spin–Boson model and the Gaussian noise model is negligible at high jump rates, indicating that the Spin–Boson model effectively replicates Gaussian noise under these conditions. It should be noted that over time, the results of the two models diverge at all speeds, and the difference between these two models is significant in longer times. According to Fig. 6, at higher speeds, such as $1\times 10^{8}s^{-1}$, the Spin–Boson model and Gaussian noise model exhibit very good agreement. Therefore, the Spin–Boson model can effectively mimic the Gaussian noise model for the ion channel at high speeds. The result of this work is in full agreement with recent discoveries. In a recent study^33^, the relationship between the rate of hopping and maintaining coherence in ion channels was investigated and it was found that the system is coherent in ion channels with high throughput rates. The present work also provides further evidence as to why ion channels have a high selectivity rate.Our numerical results suggest that the decoherence formalism can be a valuable tool for exploring the paradox of selectivity and high speed in ion channels. This approach, which has been successfully applied to various biological systems, can similarly be extended to study ion channels. The ability of the decoherence model to effectively mimic the classical noise model indicates that coherence effects may play a significant role in the ion channel’s function. Specifically, the high selectivity and rapid ion transport observed in these channels could be underpinned by quantum mechanical principles, offering a new perspective on their operation. By employing the decoherence formalism, we can potentially uncover quantum mechanisms that govern the efficient and selective ion transport, akin to how decoherence has elucidated other biological phenomena such as olfaction. This insight not only enhances our understanding of ion channels but also opens up new avenues for future research to identify specific quantum interactions that contribute to their functionality. It is important to note, however, that the implications of our study may vary depending on the nature of the biological system under investigation. Differences may arise when applying decoherence to ion channels compared to other systems, like olfactory receptors, highlighting the need for further research to explore these distinctions comprehensively.

It should be noted that for values of $\gamma$ and $\omega$ different from those considered here, the Spin–Boson and Gaussian noise models show good agreement at high speeds. Therefore, the loss of coherence described by the classical noise formalism in the potassium ion channel, which is characterized by Gaussian noise, can be well described by the Spin–Boson model, and these two dynamics are in good agreement. For the mechanisms that ignore the presence of water molecules, the two models have a good match.

## Conclusion

The purpose of this article is to investigate the behavior of potassium ion channels by two models: Spin–Boson and classical noise models. Recently, it has been suggested that quantum coherence is part of the ion selection process. But due to the biological temperature and the coupling of the channels with the environment, the system loses its coherence. When the system interacts with its environment, it goes through a decoherence process. In various works, the loss of coherence caused by environmental entanglement has been compared with classical oscillations that cause system disturbances. In other words, classic noise processes are used to simulate the effect of the environment on the system. This is usually done by adding random terms to the Hamiltonian of the system. The question raised here is whether, in principle, the quantum approach is reasonable for us to use in ion channel systems. For this purpose, after the possible hypothesis of maintaining coherence in ion channels, we compared the quantum approach and the classical noise model in this paper. We compared the quantum decoherence that occurs when a single quantum system is entangled with environmental degrees of freedom with the apparent decoherence obtained by averaging over a set of unitary evolutions produced by a stochastic Hamiltonian in an ion channel system. To investigate the behavior of the ion channel system, we assumed our model consists of the superposition of two states for the system: states 1,3, and 2,4. In the first one, potassium ions are in the 1st and 3rd positions and the 2nd and 4th positions are occupied by water molecules. In the second case, positions 2 and 4 are occupied by potassium ions, and positions 1 and 3 are occupied by water molecules. We studied this process using two models, Spin–Boson and classical noise, considering two types of Gaussian noise and Ornstein-Uhlenbeck noise, and solved the equations numerically. At first, this system was considered as a two-level one, which is coupled to the protein environment, which includes a set of coordinated oscillators. The effects of the environment on the system are briefly described by the spectral density. The behaviour of this system was investigated by the Spin–Boson model with tunneling. The master Eq. (10) was solved and investigated in different regimes. Then, the classical noise model was used to investigate the behavior of the ion channel system. We used two common types of classical noise, Gaussian noise and Ornstein-Uhlenbeck noise, in our model to evaluate the classical noise framework. We have shown in what conditions the decoherence caused by the interaction of the ion channel system with its environment can simulate classical noise effects. The results showed that at high hopping rates, the loss of coherence caused by a white Gaussian noise can be well described by the Spin–Boson model considering an ohmic environment with high temperature. By reducing the speed of passage, we can clearly see how the results of quantum decoherence differ from the classical noise model for Gaussian noise. In examining the compatibility of the Ornstein-Uhlenbeck noise model with the Spin–Boson model, it was found that this noise model is not well-suited for comparing with quantum decoherence in our system. The reason is that ion channel interactions are sharp, particularly at high channel speeds, and we anticipate no environmental changes resulting from these interactions. Therefore, the Markov assumption is unlikely to hold in this context. Because the channel interactions are sharp, we expect the noise distribution to be sharp as well. Therefore, Gaussian distributions, known for their stability and constancy over time, emerge as the preferred criterion in our ion channel analysis. Based on this criterion, the higher the speed of ion passage in the channel, the more compatible the classical noise model and the Spin–Boson model are. We showed the predictions of the decoherence theory were close enough to the classical noise model used in ion channels. Based on our results, it may be possible to investigate these systems further utilizing the decoherence model. For future work, we can look for a convincing theory and mechanism for the ion channel and investigate the role of quantum coherence in the selectivity process. This mechanism should be able to explain well how an ion channel allows only one specific ion to pass through and rejects all ions of smaller diameters.

## Data Availability

The datasets used and/or analyzed during the present study are available from the corresponding author upon reasonable request.

## References

[1] Pardo LA (2004). Voltage-gated potassium channels in cell proliferation. Physiology.

[2] Westra RL (2019). Resonance-driven ion transport and selectivity in prokaryotic ion channels. Phys. Rev. E.

[3] O’Grady SM, Lee SY (2003). Chloride and potassium channel function in alveolar epithelial cells. Am. J. Physiol.-Lung Cell. Mol. Physiol..

[4] Qi S (2021). Foldamer-based potassium channels with high ion selectivity and transport activity. J. Am. Chem. Soc..

[5] Corry B, Chung S-H (2006). Mechanisms of valence selectivity in biological ion channels. Cell. Mol. Life Sci. CMLS.

[6] Noskov SY, Berneche S, Roux BÎ (2004). Control of ion selectivity in potassium channels by electrostatic and dynamic properties of carbonyl ligands. Nature.

[7] Sokolova O, Ludmila K-P, Nikolaus G (2001). Three-dimensional structure of a voltage-gated potassium channel at 2.5 nm resolution. Structure.

[8] Berneche S, Roux B (2001). Energetics of ion conduction through the $$\text{K}^{+}$$ channel. Nature.

[9] Doyle DA (1998). The structure of the potassium channel molecular basis of $$\text{ K}^{+}$$ conduction and selectivity. Science.

[10] Allen TW (2000). The potassium channel: Structure, selectivity and diffusion. J. Chem. Phys..

[11] Salari V (2015). On the classical vibrational coherence of carbonyl groups in the selectivity filter backbone of the KcsA ion channel. J. Integr. Neurosci..

[12] Salari V (2015). Quantum decoherence time scales for ionic superposition states in ion channels. Phys. Rev. E.

[13] Dudev T, Lim C (2009). Determinants of $$ K^{+} $$ vs $$ Na^{+} $$ selectivity in potassium channels. J. Am. Chem. Soc..

[14] Thompson AN (2009). Mechanism of potassium-channel selectivity revealed by $$ Na^{+} $$ and $$ Li^{+} $$ binding sites within the KcsA pore. Nat. Struct. Mol. Biol..

[15] Allen TW, Kuyucak S, Chung S-H (1999). Molecular dynamics study of the KcsA potassium channel. Biophys. J ..

[16] Biggin PC (2001). Potassium and sodium ions in a potassium channel studied by molecular dynamics simulations. Biochimica et Biophysica Acta (BBA)-Biomembranes.

[17] Domene C, Grottesi A, Sansom MSP (2004). Filter flexibility and distortion in a bacterial inward rectifier $$ K^{+} $$ channel: simulation studies of KirBac1. 1. Biophys. J..

[18] Shrivastava IH, Sansom MSP (2000). Simulations of ion permeation through a potassium channel: Molecular dynamics of KcsA in a phospholipid bilayer. Biophys. J ..

[19] Burykin Anton, Kato Mitsunori, Warshel Arieh (2003). Exploring the origin of the ion selectivity of the KcsA potassium channel. Prot. Struct. Funct. Bioinform..

[20] Allen TW, Chung SH (2001). Brownian dynamics study of an open-state KcsA potassium channel. Biochimica et Biophysica Acta (BBA)-Biomembranes.

[21] Roux BÎ (2005). Ion conduction and selectivity in $$ K^{+} $$ channels. Annu. Rev. Biophys. Biomol. Struct..

[22] Marais A (2018). The future of quantum biology. J. R. Soc. Interface.

[23] Ghasemi F, Shafiee A (2020). An investigation into the energy transfer efficiency of a two-pigment photosynthetic system using a macroscopic quantum model. Biosystems.

[24] Kim Y (2021). Quantum biology An update and perspective. Quant. Rep..

[25] Ghasemi F, Shafiee A (2019). A quantum mechanical approach towards the calculation of transition probabilities between DNA codons. Biosystems.

[26] Tirandaz A, Ghahramani FT, Shafiee A (2014). Emergence of molecular chirality due to chiral interactions in a biological environment. J. Biol. Phys..

[27] Scholes GD (2017). Using coherence to enhance function in chemical and biophysical systems. Nature.

[28] Lambert N (2013). Quantum biology. Nat. Phys..

[29] Engel GS (2007). Evidence for wavelike energy transfer through quantum coherence in photosynthetic systems. Nature.

[30] Slocombe L, Sacchi M, Al-Khalili J (2022). An open quantum systems approach to proton tunnelling in DNA. Commun. Phys..

[31] Mohseni, M. *et al.* (eds) *Quantum Effects in Biology* (Cambridge University Press, 2014).

[32] Ganim Z, Tokmakoff A, Vaziri A (2011). Vibrational excitons in ionophores: Experimental probes for quantum coherence-assisted ion transport and selectivity in ion channels. New J. Phys..

[33] Seifi M, Soltanmanesh A, Shafiee A (2022). Quantum coherence on selectivity and transport of ion channels. Sci. Rep..

[34] Vaziri A, Plenio MB (2010). Quantum coherence in ion channels: Resonances, transport and verification. New J. Phys..

[35] Salari V, Naeij H, Shafiee A (2017). Quantum interference and selectivity through biological ion channels. Sci. Rep..

[36] Summhammer J, Sulyok G, Bernroider G (2020). Quantum mechanical coherence of f $$\text{ K}^{+}$$ ion wave packets increases conduction in the KcsA ion channel. Appl. Sci..

[37] Summhammer J, Salari V, Bernroider G (2012). A quantum-mechanical description of ion motion within the confining potentials of voltage-gated ion channels. J. Integr. Neurosci..

[38] Cifuentes AA, Semiao FL (2014). Quantum model for a periodically driven selectivity filter in a $$\text{ K}^{+}$$ ion channel. J. Phys. B: At. Mol. Opt. Phys..

[39] Gu B, Franco I (2019). When can quantum decoherence be mimicked by classical noise?. J. Chem. Phys..

[40] Costa-Filho JI (2017). Enabling quantum non-Markovian dynamics by injection of classical colored noise. Phys. Rev. A.

[41] Chenu A (2017). Quantum simulation of generic many-body open system dynamics using classical noise. Phys. Rev. Lett..

[42] Yang W, Ma W-L, Liu R-B (2016). Quantum many-body theory for electron spin decoherence in nanoscale nuclear spin baths. Rep. Prog. Phys..

[43] Budini AA (2001). Quantum systems subject to the action of classical stochastic fields. Phys. Rev. A.

[44] Szańkowski PC (2020). Noise representations of open system dynamics. Sci. Rep..

[45] Crow D, Joynt R (2014). Classical simulation of quantum dephasing and depolarizing noise. Phys. Rev. A.

[46] Ma W-L (2015). Classical nature of nuclear spin noise near clock transitions of Bi donors in silicon. Phys. Rev. B.

[47] Saira O-P (2007). Equivalent qubit dynamics under classical and quantum noise. Phys. Rev. A.

[48] Schneider S, Milburn GJ (1998). Decoherence in ion traps due to laser intensity and phase fluctuations. Phys. Rev. A.

[49] Breuer, H-P. & Petruccione, F. *The Theory of Open Quantum Systems* (Oxford University Press on Demand, 2002).

[50] de León-Montiel R, Torres JP (2013). Highly efficient noise-assisted energy transport in classical oscillator systems. Phys. Rev. Lett..

[51] Erich, J. *et al.**Decoherence and the Appearance of a Classical World in Quantum Theory* (Springer, 2013).

[52] Marcinkiewicz J (1939). Sur une propriété de la loi de Gauss. Math. Z..

[53] Kiely A (2021). Exact classical noise master equations: Applications and connections. Europhys. Lett..

[54] Budini AA (2000). Non-Markovian Gaussian dissipative stochastic wave vector. Phys. Rev. A.

[55] Uhlenbeck GE, Ornstein LS (1930). On the theory of the Brownian motion. Phys. Rev..

[56] Salari, V. *et al.* Plausibility of quantum coherent states in biological systems. *J. Phys. Conf. Ser.* **306**(1) (2011).

[57] Mohammadi A, Shafiee A (2024). Quantum non-Markovianity, quantum coherence and extractable work in a general quantum process. Phys. Chem. Chem. Phys..

[58] Naeij, H. R. Rotational decoherence due to thermal photon scattering. *arXiv preprint* arXiv:2210.06133 (2022).

[59] Carlesso M, Naeij HR, Bassi A (2021). Perturbative algorithm for rotational decoherence. Phys. Rev. A.

[60] Schlosshauer, M. A. *Decoherence: And the Quantum-to-Classical Transition* (Springer, 2007).

[61] Naeij HR, Shafiee A (2020). Langevin equation for a dissipative macroscopic quantum system: Bohmian theory versus quantum mechanics. Quant. Stud. Math. Found..

[62] Berneche S, Roux BÎ (2003). A microscopic view of ion conduction through the $$\text{ K}^{+}$$ channel. Proc. Natl. Acad. Sci..

[63] Bhattacharya, S. & Roy, S. Quantum thermodynamics and coherence in ion channels. *Applied Physics, System Science and Computers: Proceedings of the 1st International Conference on Applied Physics, System Science and Computers (APSAC2016),* September 28-30, Dubrovnik, Croatia. Springer International Publishing, 2018.

[64] Gilmore JB, McKenzie RH (2006). Criteria for quantum coherent transfer of excitations between chromophores in a polar solvent. Chem. Phys. Lett..

[65] Pachón LA, Brumer P (2011). Physical basis for long-lived electronic coherence in photosynthetic light-harvesting systems. J. Phys. Chem. Lett..

[66] Tao G, Miller WH (2010). Semiclassical description of electronic excitation population transfer in a model photosynthetic system. J. Phys. Chem. Lett..

[67] Huelga SF, Plenio MB (2011). Quantum dynamics of bio-molecular systems in noisy environments. Procedia Chem..

[68] Nalbach P, Eckel J, Thorwart M (2010). Quantum coherent biomolecular energy transfer with spatially correlated fluctuations. New J. Phys..

[69] Shafiee A, Tirandaz A (2011). Comparison between decoherence time for a two-state spin 1/2 system with its corresponding quantum retrieval period. Open Phys..

[70] Soltanmanesh A, Shafiee A (2019). Clausius inequality versus quantum coherence. Eur. Phys. J. Plus.

[71] Soltanmanesh A, Naeij HR, Shafiee A (2020). Can thermodynamic Behavior of Alice’s Particle Affect Bob’s particle?. Sci. Rep..

[72] Soltanmanesh, A. & Shafiee, A. Quantum Decoherence in System-Bath Interferometry. *arXiv preprint* arXiv:1802.07468 (2018).

[73] Roux B, Schulten K (2004). Computational studies of membrane channels. Structure.

[74] Corry B (2018). The naked truth about $$\text{ K}^{+}$$ selectivity. Nat. Chem..

[75] Roux B (2002). Theoretical and computational models of ion channels. Curr. Opin. Struct. Biol..

[76] Kratochvil HT (2016). Instantaneous ion configurations in the $$\text{ K}^{+}$$ ion channel selectivity filter revealed by 2D IR spectroscopy. Science.

[77] Hodgkin AL, Keynes RD (1955). The potassium permeability of a giant nerve fibre. J. Physiol..

[78] Zhou Y, MacKinnon R (2003). The occupancy of ions in the K+ selectivity filter: Charge balance and coupling of ion binding to a protein conformational change underlie high conduction rates. J. Mol. Biol..

